# Quicker Exogenous Orienting and Slower Endogenous Orienting in Autistic People

**DOI:** 10.1007/s10803-024-06311-8

**Published:** 2024-03-06

**Authors:** Shuting Li, Keitaro Machida, Emma L. Burrows, Katherine A. Johnson

**Affiliations:** 1 Melbourne School of Psychological Sciences, The University of Melbourne, Parkville, VIC 3010 Australia; 2 Florey Institute of Neuroscience and Mental Health, The University of Melbourne, Parkville, VIC 3052 Australia

**Keywords:** Autism, Exogenous orienting, Endogenous orienting, Posner task, Pupillary response

## Abstract

Research is equivocal on whether attention orienting is atypical in autism. This study investigated two types of attention orienting in autistic people and accounted for the potential confounders of alerting level, co-occurring symptoms of attention-deficit/hyperactivity disorder (ADHD) and anxiety, age, and sex. Twenty-seven autistic participants (14 males; 9–43 years) and 22 age- and sex-matched non-autistic participants (13 males; 9–42 years) completed the exogenous and endogenous Posner tasks. Response time and pupillometric data were recorded. Autistic participants were faster at orienting attention to valid cues in the exogenous task and slower at disengaging from invalid cues in the endogenous task compared to non-autistic participants. With increasing age, autistic participants showed faster exogenous and endogenous orienting, whereas non-autistic participants showed faster exogenous orienting but stable speed of endogenous orienting. Higher ADHD symptoms were associated with slower exogenous orienting in both groups, whereas higher anxiety symptoms were associated with faster exogenous orienting only in autistic participants. No group differences were noted for alerting levels, sex, or pupillary responses. This study provides new evidence of superior exogenous orienting and inefficient endogenous orienting in autistic people and suggests that age and co-occurring symptoms are important to consider when assessing attention orienting in autism.

Spatial attention orienting is an essential cognitive function that determines where we focus our concentration. Atypical attention orienting has been suggested as a promising early indicator of autism spectrum disorder (ASD) prior to the emergence of socio-communicative deficits (Baranek et al., 2018; Canu et al., 2021; Elsabbagh et al., 2013). When attention orienting is assessed experimentally in autistic people, however, findings are inconsistent (Keehn et al., 2013; Landry & Parker, 2013). It remains unclear which experimental and individual factors may trigger or intensify atypical attention orienting in ASD.

Attention orienting can be controlled by exogenous (i.e., stimulus-driven) or endogenous (i.e., goal-driven) processes (Corbetta & Shulman, 2002), both of which can be measured by variations of the Posner task (Posner, 1980). In this task, participants are asked to use cues to orient their attention to the location of an upcoming target that appears at either the left or right side of screen. In the exogenous task, the cue is non-symbolic, such as a peripheral light flash, and does not predictive the target location. In the endogenous task, the cue is typically symbolic, such as an arrow or eye gaze in the middle of screen, and is predictive. Cues that mix features of exogenous and endogenous cues or that have been overlearned, such as arrows, engage processes independent of exogenous and endogenous orienting (Ristic & Kingstone, 2012; Vecera & Rizzo, 2006) or engage both exogenous and endogenous orienting (Losier & Klein, 2001; Warner et al., 1990), making it difficult to interpret the findings.

Previous research using the Posner task has yielded inconsistent results in autistic people. This may be due to variation in the types of cues used. Early studies of exogenous orienting suggested that autistic people showed delayed or deficient orienting (Casey et al., 1993; Harris et al., 1999; Townsend, Courchesne et al., 1996; Townsend, Harris et al., 1996), but these studies used predictive peripheral cues, which appear to engage both exogenous and endogenous orienting (Warner et al., 1990). In subsequent studies that used nonpredictive peripheral cues, some observed intact orienting (Iarocci & Burack, 2004; Pruett et al., 2011) while others found slower orienting (Ronconi et al., 2018) in autistic people. These studies, however, either used complex cues (e.g., with varied sizes; Ronconi et al., 2018) or introduced distracting stimuli (Iarocci & Burack, 2004) that may induce extra cognitive processing other than a stimulus-driven reaction.

Results of endogenous orienting studies have also been mixed; some studies reported intact orienting in autistic people (Pruett et al., 2011; Renner et al., 2006) while others reported difficulties in disengaging attention (Flanagan et al., 2015; Tsai et al., 2011). These studies of endogenous orienting all employed overlearned cues used in daily life, such as arrows and eye-gaze, that may confound the measurement of endogenous orienting. To date, it remains unknown whether autistic people show impaired endogenous orienting based on non-social rule-based cues.

Several unaccounted factors may help to explain these previous inconsistent findings. First, varying levels of arousal may affect orienting of attention (Callejas et al., 2004; Spagna et al., 2014). Presenting an alerting cue was found to improve the accuracy or speed of response in autistic participants (Fan et al., 2012; Hames et al., 2016; Mash et al., 2020), but how alerting levels affect the process of attention orienting in autistic people remains unclear. Physiological measures of pupil dilation can be used to capture changes in alertness (Aston-Jones et al., 2007; Carter et al., 2013; Yang et al., 2021). Two studies have measured pupillary responses in autistic people using modified Posner tasks and both suggested hyper-alertness in autism (Aldaqre et al., 2016; Boxhoorn et al., 2020). Aldaqre et al. (2016) found that pupil dilations were larger in autistic people when orienting was based on high-communicative versus low-communicative social cue (i.e., pointing versus grasping gestures), which was not found in non-autistic people. Boxhoorn et al. (2020) found that autistic people who showed larger pupil dilations were slower at orienting, which was also not found in non-autistic people. These studies, however, included settings that may affect pupillary measurement, with one requiring interpretation of different social cues and shifting gaze (Aldaqre et al., 2016) and the other requiring discrimination between distractor and target (Boxhoorn et al., 2020). Therefore, it remains inconclusive how alertness levels affect orienting and how pupil alertness changes during attention orienting in autism.

Second, co-occurring symptoms in autism may also confound orienting results. Attention-deficit/hyperactivity disorder (ADHD) and anxiety are two common comorbid symptoms in autism (Lai et al., 2019) and both may affect attention orienting (Caldani et al., 2020; Ghassemzadeh et al., 2019; Ortega et al., 2013). To date, the impact of co-occurring ADHD and anxiety symptoms on attention orienting in autistic people is not clearly understood.

Third, other factors, including age and sex, may also influence attention orienting. A meta-analysis found that impairments of attention orienting in autistic people increased with age (O. Landry & Parker, 2013). This finding, however, remains to be confirmed as most reviewed studies included only children. No study has addressed how sex could influence attention orienting in autism, although sex differences in orienting have been shown in non-autistic people (Bayliss et al., 2005; Feng et al., 2011; Merritt et al., 2007).

This study aimed to investigate attention orienting in autistic people using the Posner task, with consideration of the effects of alertness, co-occurring anxiety and ADHD symptoms, age, and sex. To reduce ambiguous interpretations, we used non-symbolic, non-predictive, peripherally located cues to examine exogenous orienting and newly learned, centrally presented coloured cues to examine endogenous orienting (Johnson et al., 2020). It was hypothesised that (1) autistic people would show orienting deficits in performance on both exogenous and endogenous tasks, (2) the provision of an alerting tone would improve the efficiency of attention orienting in autistic people, and (3) higher anxiety and ADHD symptoms would be associated with atypical orienting in autistic people. Due to the paucity of research, no hypotheses on the effects of age and sex were proposed. As subtle changes in attention orienting may not be captured by behavioural measurements alone, the current study measured pupil dilation (PD) peak amplitude and the latency to PD peak amplitude as additional sensitive indicators of alertness. To date, no ASD studies have tested pupil responses in a conventional Posner paradigm. Therefore, no hypotheses on the pupil responses during attention orienting were proposed.

## Methods

### Participants

Twenty-nine autistic people with a clinical diagnosis of ASD and 26 non-autistic people were recruited. Inclusion criteria were a confirmed ASD diagnosis using the Autism Diagnostic Observation Schedule, Second Edition (ADOS-2; Lord et al., 2012) for autistic participants, a Raven’s 2 standard score of greater than 70 (Raven et al., 2018) and an overall accuracy performance of more than 70% on the orienting tasks. One autistic and two non-autistic participants were excluded due to low accuracy. One autistic participant refused to participate, and data of two non-autistic participants were lost due to technical issues. The final sample included 27 autistic and 22 age- and sex-matched non-autistic participants (see Table 1).

**Table 1:** Demographic Characteristics of the Participants

	Autistic^a^ (*n* = 27)	Non-Autistic (*n* = 22)	Group comparison
Sex (M:F)	14:13	13:9	*χ*^*2*^(1) = 0.26, *p* = .61
Age in years^b^, *M* (*SD*), range	22 (10), 9–43	21 (9), 9–42	*t*(47) = 0.57, *p* = .57
ADOS-2^c^ (*n*)	High symptoms (9) Moderate symptoms (14) Low symptoms (4)	-	-
Socioeconomic index^d^	1: 7%, 2: 4%, 3: 15%, 4: 33%, 5: 33%, NR: 7%	1: 5%, 2: 0%, 3: 5%, 4: 32%, 5: 50%, NR: 9%	*χ*^*2*^(5) = 3.11, *p* = .68
Raven’s 2, *M* (*SD*), range	104 (13), 76–132	112 (14), 83–146	*t*(47) = -2.03, *p* = .048
State anxiety, *M* (*SD*) (self-reported)	55 (9)	43 (8)	*t*(47) = 4.92, *p* < .001
Trait anxiety, *M* (*SD*) (self-reported)	64 (13)	50 (14)	*t*(47) = 3.60, *p* < .001
Anxiety, *M* (*SD*) (informant-reported)	29 (16)	9 (7)	*t*(47) = 5.67, *p* < .001
ADHD index, *M* (*SD*) (self-reported)	64 (12)	49 (10)	*t*(47) = 4.89, *p* < .001
ADHD index, *M* (*SD*) (informant-reported)	63 (17)	44 (8)	*t*(47) = 5.01, *p* < .001
Co-diagnoses (*n*)	anxiety (11), ADHD (5), sleeping disorder (4), depression (3), obsessive-compulsive disorder (1), dysgraphia (1), colour-blindness^e^ (1)	None	-
Psychoactive medications reported being used at time of testing (*n*)	Zoloft (4), Concerta (1), Cymbalta and Reboxetine (1), Latuda (1), Lexapro (1), Venlafaxine (1)	None	-

Note ^a^ All autistic participants spoke English fluently. ^b^ Ten autistic and 9 non-autistic participants were below 18 years old. The age range was chosen to include all interested participants. ^c^ Nine autistic participants were assessed using ADOS-2 Module 3 and 18 autistic participants were assessed using ADOS-2 Module 4. ^d^ Based on the Index of Relative Socio-economic Advantage and Disadvantage (IRSAD) of the Socio-Economic Indexes for Areas (SEIFA) 2016. 1 = most disadvantaged, 5 = most advantaged. NR = not reported. ^e^ Participant has confirmed his ability to distinguish blue and yellow endogenous cues and correctly answered the questions relating the meaning of coloured cues during the endogenous task

Autistic participants were recruited through clinics, hospitals, universities, and the local community. Non-autistic participants were recruited through the local community. Informed written consent was obtained from adult participants and from caregivers of child participants.

## Materials

### Orienting Task

**Fig. 1:**
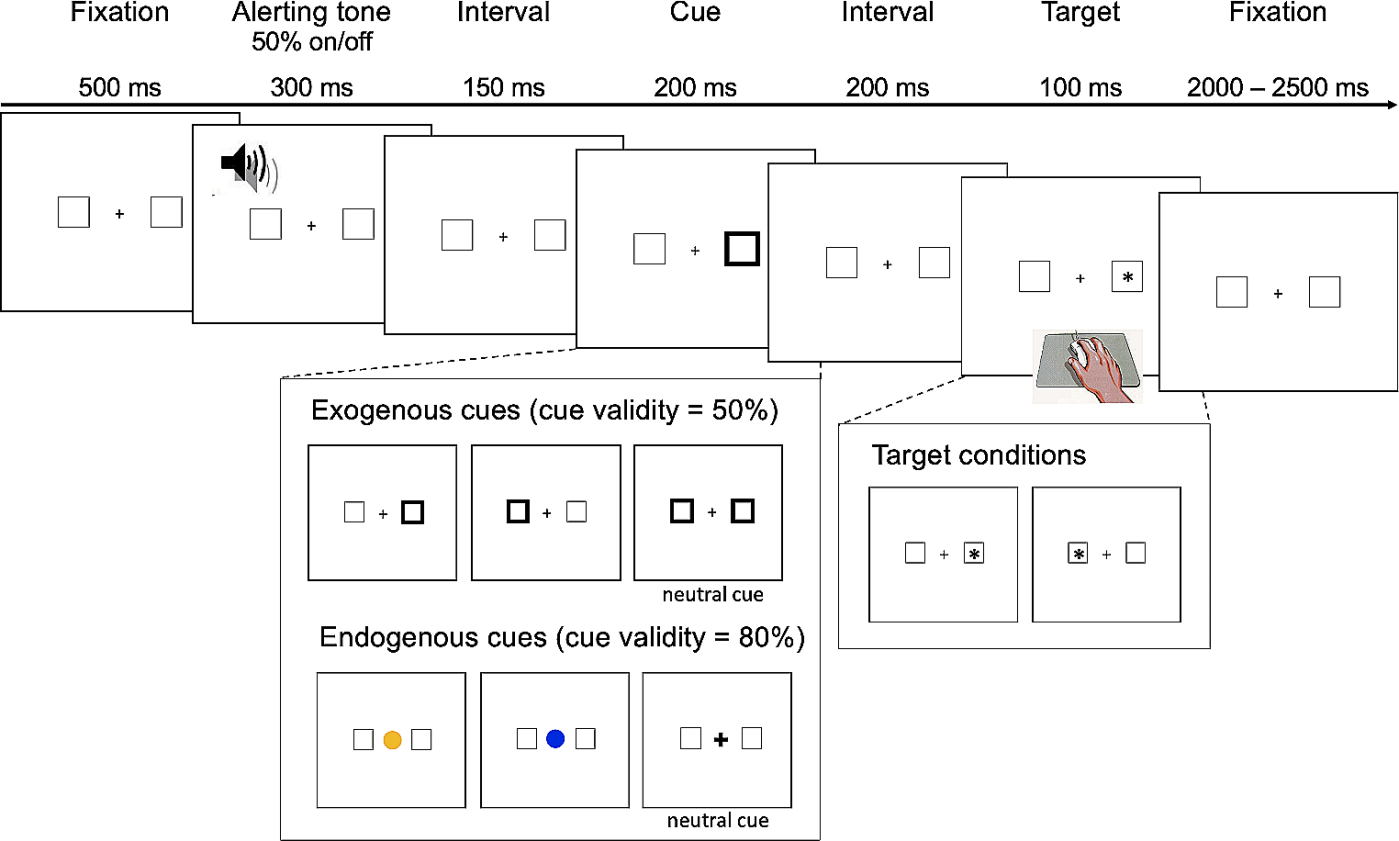
The Timeline and Stimuli Used in the Exogenous and Endogenous Tasks. *Note* In the endogenous task, the valid and invalid cues were a yellow or blue filled circle. The contingency between the colour and target location was counterbalanced in participants

The exogenous and endogenous orienting tasks (Posner, 1980) were administered using E-Prime (Psychology Software Tools, 2001) on a Tobii eye-tracking system (Fig. 1). For the exogenous task, each trial began with a background visual display of a central cross and a left and right square. Participants were instructed to fix their gaze on the central cross. In 50% of trials, a 300 ms alerting tone was presented 500 ms after the commencement of the trial, then a 150 ms interval, and a 200 ms visual cue. In the other 50% of trials, no alerting tone was presented and a visual cue appeared 950 ms after the trial commencement. There were three visual cues: valid, invalid, and neutral. Valid cues correctly indicated the location of the subsequent target. Invalid cues indicated the location opposite to the target. Neutral cues provided no spatial information about the target. In the exogenous task, the valid and invalid cues were a thickening of one peripheral square and the neutral cue was a thickening of both peripheral squares concurrently. Each cue was presented with equal probability (33.3% each). The offset of the cue was followed by a 200 ms interval and then the presentation of the target (an asterisk) in one square for 100 ms. Participants were asked to indicate the location of the target as quickly and accurately as possible by pressing either the left or right mouse button using the index or middle finger of their dominant hand, respectively. Each trial ended with the background visual display for between 2 and 2.5 s.

The endogenous task was the same except that the cues were coloured blue or yellow and centrally presented. The colour represented a rule that the participants learned during the practice block. For example, a yellow circle meant that the target was likely to appear to the left and a blue circle meant that the target would appear to the right. The colour rule was counterbalanced across participants. If the central cross thickened, this meant that the target could appear in either location. The neutral cue was designed to resemble the fixated cross and induced low cognitive demand for interpretation. The neutral cue was presented in 33% of trials, and valid and invalid cues were presented in the remaining trials with an 80:20 probability, respectively.

The exogenous task contained one 12-trial practice block and three 48-trial experimental blocks. The endogenous task contained one 12-trial practice block and four 48-trial experimental blocks. In the practice blocks, the spatial cues were 100% valid and feedback on accuracy and speed of response was provided after each response. No feedback was provided in the experimental blocks. In the endogenous task, participants answered a multiple-choice question relating to the meaning of the cues at the end of each block, with the correct answer provided immediately after a response made. These questions were presented to ensure that participants understood the association between the coloured cues and the location of the target.

### Eye Tracking

Pupil diameters were recorded at 300 Hz using a Tobii-TX300 eye tracker (Tobii Technology, Stockholm, Sweden) on a 23-inch eye-tracking monitor with resolution fixed at 1,920 × 1,080 pixels. Operating distance was a constant 65 cm from the monitor and head movements were stabilised using a chin rest. The sizes of the fixation cross, peripheral squares, and other stimuli were 0.8° × 0.8°, 2° × 2°, and 1° × 1°, respectively. Prior to commencing each orienting task, the eye tracker was calibrated using a nine-point calibration procedure.

### Raven’s 2 Progressive Matrices

Non-verbal general cognitive ability was assessed using the Raven’s 2 digital short form (Raven’s 2 DSF: Raven et al., 2018), with the purpose of excluding any participants with a score of less than 70. We wanted to ensure that participants had the cognitive ability to understand the instructions for the attention orienting tasks. On each trial, participants were shown a geometric pattern matrix with a piece missing and an array of shape options. Participants used the computer mouse to choose the correct shape to complete the pattern. The number of items answered correctly was summed, and age-adjusted standardised scores were generated. This assessment measures skills of observation, thinking, and general intellectual capacity while minimising the influence of language and cultural differences. Raven’s 2 DSF was reported to have good reliability (IRT-based marginal reliability = .8) and reasonable correlations with previous Raven’s tests and other cognitive ability instruments (corrected *r* = .4–.8, depending on the similarity of constructs; Raven et al., 2018).

### Anxiety Assessment

Child (aged under 18 years) and adult participants (aged 18 years and older) were assessed using the State-Trait Anxiety Inventory for Children (STAI-CH; Spielberger, 1973) and the State-Trait Anxiety Inventory for Adults (STAI-AD; Spielberger, 1983), respectively. The state and trait anxiety scores were standardised based on age-appropriate norms. Both inventories were received from Mind Garden Inc. (Menlo Park, USA) and demonstrated good reliability (corrected *α*: STAI-CH > .7; STAI-AD > .8) and construct validity (details reported in the STAI manuals; Spielberger, 1973; Spielberger et al., 1983).

Informant-reported anxiety symptoms were measured using the Spence Children’s Anxiety Scale-Parent (SCAS-P; Spence, 1999) and a modified version of SCAS-P for adult participants (Gillott & Standen, 2007). The SCAS-P has strong reliability (corrected *α* > .8) and high convergent validity (*r* = .6) and divergent validity (*r* = .3) compared to other related measures (Nauta et al., 2004).

### ADHD Symptoms

Self-reported ADHD symptoms were measured using the self-report Conners-3 Questionnaire (Conners, 2008) in children and the self-report Conners Adult ADHD Rating Scales (CAARS-S:L; Conners et al., 1999) in adults.

Informant-reported ADHD symptoms were measured using the parent-report Conners-3 Questionnaire (Conners, 2008) in children and the observer-report Conners Adult ADHD Rating Scales (CAARS-O:L; Conners et al., 1999) in adults.

Standardised self-reported and informant-reported ADHD index scores were calculated based on age-appropriate norms. All these questionnaires showed strong reliability (*α* > .8) and good construct validity (details reported in the Conners-3 and CAARS manuals; Conners, 2008; Conners et al., 1999).

### Procedures

Autistic participants were assessed with the ADOS-2 (Lord et al., 2012) by trained administrators in a separate session prior to the testing session. In the 1.5 h testing session, participants completed the Posner tasks, Raven’s 2, and two questionnaires relating to their anxiety and ADHD symptoms. The administration order of the tasks and questionnaires were counterbalanced. Informants of participants were invited to complete two questionnaires on the anxiety and ADHD symptoms of the participants.

### Data Preparation and Statistical Analysis

Analyses were conducted separately for the exogenous and endogenous tasks. Behavioural data were analysed using STATA (StataCorp., College Station, TX, USA), and pupillometric data were analysed using both STATA and Matlab (Mathworks, Natick, MA, USA).

### Behavioural Data Preparation

Response time (RT) between target presentation and button press was recorded. Responses prior to target onset or quicker than 100 ms were regarded as anticipation errors. No response or responses slower than 2000 ms were regarded as omission errors. Clicking on the incorrect mouse side was regarded as an incorrect response. Calculations relating to RT only included correct trials. For each participant, mean RT, accuracy (number of correct responses/total trials), counts of each type of error, and three indices of attention orienting - the validity effect (invalid − valid trials RT), benefit effect (neutral − valid trials RT), and cost effect (invalid − neutral trials RT) were calculated. The validity effect comprehensively captures the attention orienting process, including disengaging, shifting and engaging (Fan et al., 2009). The benefit effect measures the benefit of anticipating the likely location of the target on attention shifting and engaging. The cost effect measures the efficiency of disengaging attention from the incorrect to correct target location induced by invalid cue.

### Pupillometric Data Preparation

Missing pupillometric data were expected when participants blinked or looked away from the monitor. For each eye, trials with more than 50% missing data were excluded. Participants with more than 50% missing trials in either eye were excluded. Missing data were linearly interpolated. Based on this criterion, in the exogenous task, one non-autistic participant and two autistic participants were excluded from the analyses of the pupillometric data. In the endogenous task, one autistic participant was excluded from the analyses of the pupillometric data.

Baseline pupil size for each eye was calculated as the average pupil size of the initial 500 ms period of each trial prior to the onset of any stimuli. Time zero was set at the end of the baseline period. Phasic pupil size was calculated by subtracting the baseline value from the pupil sizes during the trial for each eye separately, and then an average was derived of the left and right eye data per trial. Two dependent variables were then calculated: (1) pupil dilation (PD) peak amplitude - the maximum phasic pupil size per trial, and (2) latency to the PD peak - the time difference between time zero to the maximum phasic pupil size per trial. The final included trials in the autistic and non-autistic group were not significantly different (Supplementary Table 1).

### Behavioural Data Statistical Analysis

Results were presented as unstandardised beta coefficients, *p*-values, and 95% confidence intervals. Only correct trials were included in statistical analyses. A series of linear mixed models (LMMs) were conducted with participant as a random effect. Validity effect, benefit effect, and cost effect were entered as dependent variables. In the base model, effects of group (autistic, non-autistic), tone condition (tone, no tone), age, and two-way interactions involving group (i.e., Group × Tone, Group × Age) on each of the orienting indices were examined. In the exploratory model, sex, self-reported state- and trait-anxiety, and ADHD index and their interactions with group were subsequently added to the LMMs for each orienting index. To further understand the three indices, the LMMs were rerun with RT as the dependent variables in the valid, neutral, invalid trials. No multicollinearity among the predictors was detected according to pairwise correlations (all *r*s < .90; Tabachnick & Fidell, 2013) and the variance inflation factor (VIF) (all VIFs < 10; Hair et al., 2010).

### Pupillometric Data Statistical Analysis

Results were presented as unstandardised beta coefficients, *p*-values, and 95% confidence intervals. The PD peak amplitude and the latency to the PD peak were entered as dependent variables in LMMs with participant as a random effect. In the base model, each analysis included the main effects of group (autistic, non-autistic), dummy-coded cue condition (valid, invalid, neutral), tone condition (tone, no tone), and age, two-way interactions between group and tone condition, between group and age, and between tone and cue condition, and a three-way interaction between group, tone, and cue condition. In the exploratory model, sex, self-reported state- and trait-anxiety and ADHD index and their interactions with group were subsequently added to the LMMs. To explore the group difference in phasic pupil size across the entire trial, cluster-based permutation tests were conducted with 5000 resamples in the valid and invalid trials with no alerting tone (Maris & Oostenveld, 2007; Supplementary Materials).

### Supplementary Analysis

As the autistic group was found to show a lower mean Raven’s 2 score than the non-autistic group, the exploratory models were re-run with the inclusion of Raven’s 2 scores to examine the potential effects of this group difference. Inclusion of the Raven’s 2 scores did not alter the pattern of statistical significance of the current results. The effects of Raven’s scores are presented in the Supplementary Table 2.

## Results

The groups did not differ on sex or age (Table 1). The autistic group scored lower on the Raven’s 2 and higher on all anxiety and ADHD measures than the non-autistic group (Table 1).

### Exogenous Task

#### Behavioural Data–Overall Performance

Response time, accuracy, and the number of different errors in the autistic and non-autistic group were reported in Table 2. As both groups made few errors across different cue conditions, the following analyses were conducted using response time.

**Table 2:** Response Time, Accuracy, and Number of Errors in the Autistic and Non-Autistic Groups in the Exogenous Task

Variable	Autistic M (SD)	Non-autistic M (SD)
Response Time (ms)		
Valid	366 (82)	396 (105)
Neutral	389 (121)	387 (105)
Invalid	434 (158)	422 (128)
Accuracy (%)		
Valid	96 (6)	97 (5)
Neutral	96 (6)	99 (3)
Invalid	92 (12)	96 (4)
Incorrect responses	5 (6)	3 (3)
Omission errors	1 (4)	0 (0)
Anticipation errors	2 (2)	1 (2)

### Behavioural Data–Base Model

#### Group

**Fig. 2:**
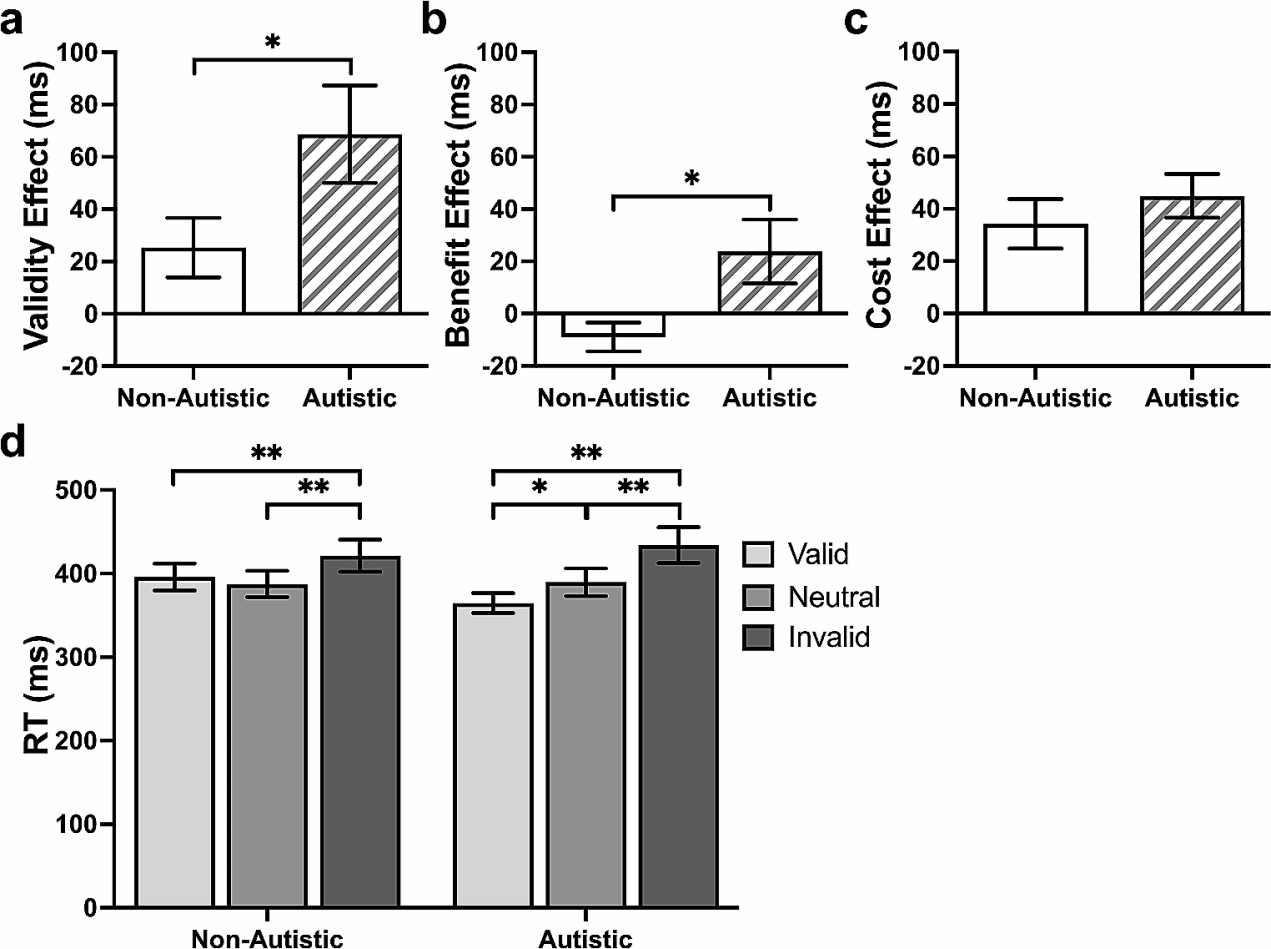
Cueing Effects and Mean Response Time (RT) in the Exogenous Task. *Note* (**a**) Significant group difference in validity effect (invalid − valid trials RT). (**b**) Significant group difference in benefit effect (neutral − valid trials RT). (**c**) Non-significant group difference in cost effect (invalid − neutral trials RT). (**d**) Differences of RT between cue conditions in each group. Data are expressed as mean ± standard errors. *p < .05, **p < .001

The autistic group showed a larger validity effect (*B* = 47.91, *p* = .03, [5.64, 90.19]; Fig. 2a) and benefit effect (*B* = 34.66, *p* = .02, [6.63, 62.70]; Fig. 2b), but a similar cost effect (*B* = 13.10, *p* = .25, [-9.17, 35.37]; Fig. 2c), than the non-autistic group. Response times were similar between groups in the valid (*p* = .30), invalid (*p* = .65), and neutral (*p* = .72) trials. In the within-group analyses, both groups responded more slowly in the invalid compared to the neutral and valid trials (both *p*s < 0.001), but only autistic participants showed a difference between the neutral and valid trials (*p* = .01; Fig. 2d).

#### Alerting Tone

**Fig. 3:**
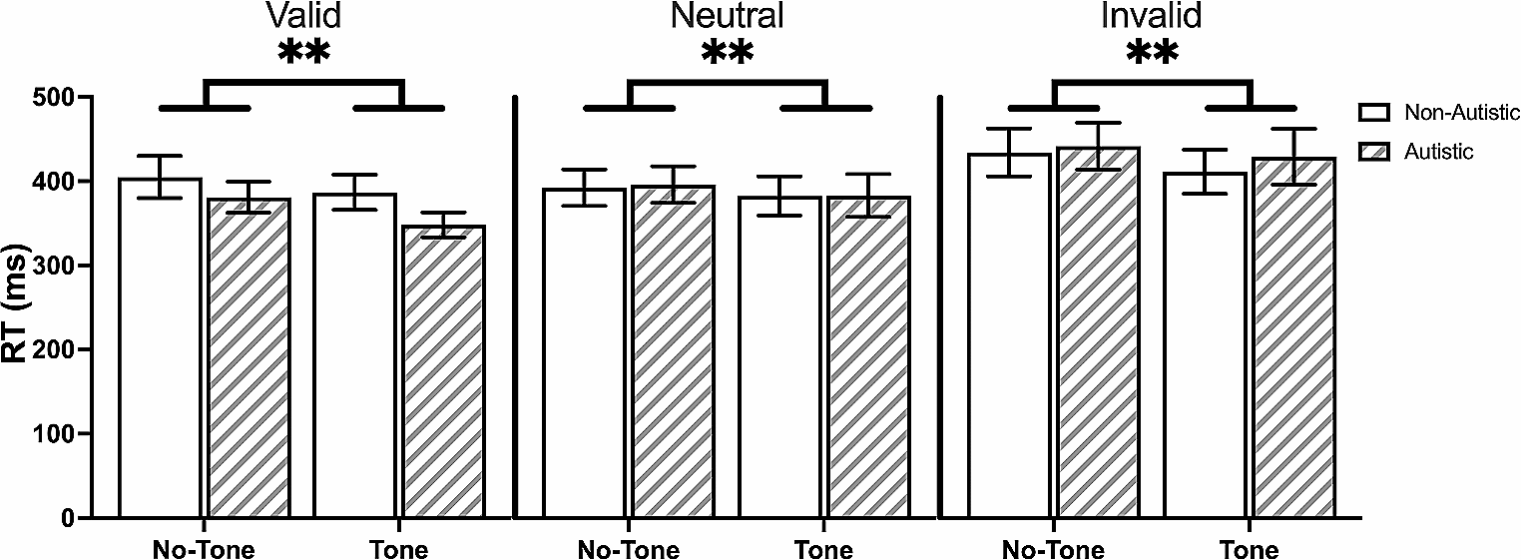
Effects of Alerting Tone on Mean Response Time (RT) in the Exogenous Task. *Note* The alerting tone led to a speeding of response time in the exogenous task, regardless of cue condition and group. Solid-coloured bars represent non-autistic group. Striped bars represent autistic group. Data are expressed as mean ± standard errors. **p < .001

The alerting tone did not affect the validity effect (*B* = 9.10, *p* = .37, [-10.37, 28.93]), the benefit effect (*B* = 15, *p* = .14, [-4.83, 34.83]), or the cost effect (*B* = -5.77, *p* = .20, [-14.66, 3.12]). The alerting tone led to a speeding in response time (*B* = -18.3, *p* < .001, [-28.6, -8.04]), in a similar manner across the different cue conditions and between groups (Fig. 3).

#### Age

The validity (*B* = -2.52, *p* = .03, [-4.77, -0.28]) and cost effects (*B* = -1.69, *p* = .01, [-2.88, -0.51]) decreased with age. No age effect was found on the benefit effect (*B* = -0.83, *p* = .28, [-2.32, -0.66]). Response times were quicker with increasing age (*B* = -5.61, *p* < .001, [-8.57, -2.65]), similarly across the different cue conditions. No group differences were found.

### Behavioural Data–Exploratory Model

#### ADHD Symptoms

Higher ADHD symptoms were associated with larger validity (*B* = 2.66, *p* = .02, 95% CI = [0.51, 4.80]) and cost effects (*B* = 1.70, *p* = .003, [0.58, 2.82]) but did not affect the benefit effect (*B* = 0.96, *p* = .20, [-0.50, 2.42]) for both groups. Response times in the valid (*p* = .01), neutral (*p* = .01), and invalid trials (*p* < .001) were slower with higher ADHD symptoms.

#### Anxiety Symptoms

**Fig. 4:**
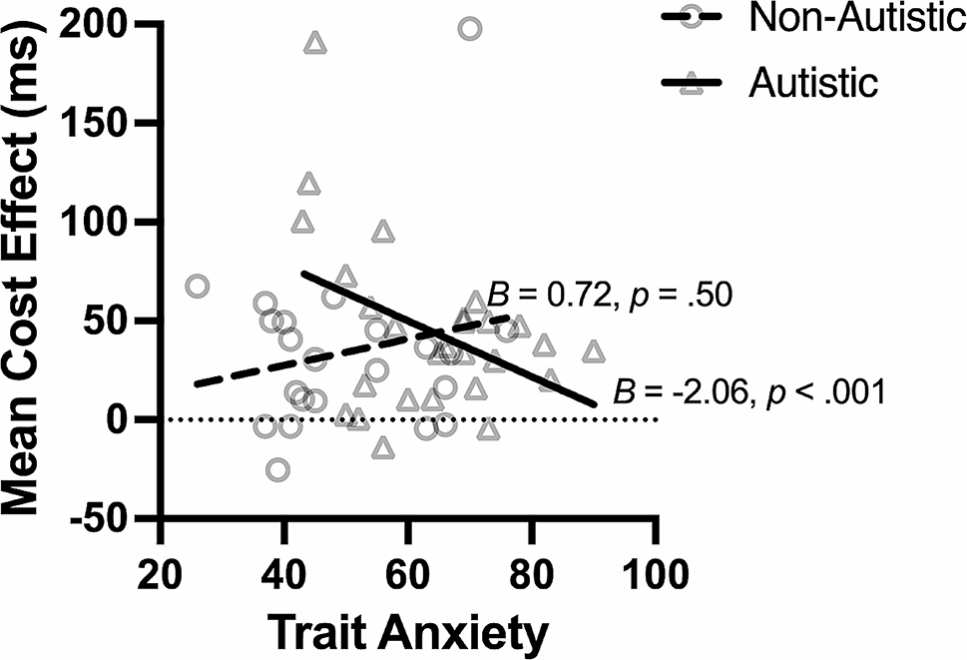
Relationship Between Cost Effect and Trait Anxiety in the Exogenous Task. *Note* The cost effect decreased with higher trait anxiety in the autistic group (solid line), but not in the non-autistic group (dashed line). The circle represents individual non-autistic participants. The triangle represents individual autistic participants

Trait anxiety affected the cost effect differently between the groups (Group × Trait Anxiety: *B* = -2.78, *p* = .02, [-5.16, -0.4]). For the autistic group, the cost effect decreased with higher trait anxiety (*B* = -2.06, *p* < .001, [-3.39, -0.73]; Fig. 4), with quicker response times in both the invalid and neutral trials (both *p*s < 0.001). For the non-autistic group, no significant effect of trait anxiety on cost effect was found (*B* = 0.72, *p* = .50, [-1.35, 2.80]). No effects of trait anxiety on orienting or benefit effects were found. No effect of state anxiety on any of the outcome variables was found.

#### Sex

Sex did not affect any of the outcome variables and did not affect the groups differentially.

### Pupillometric Data–Base Model

Pupil dilation (PD) peak amplitude was higher in the tone compared with no-tone condition (*B* = 0.036, *p* < .001, [0.028, 0.045]). There were no other significant effects.

The latency to PD peak did not differ according to group, cue condition, tone condition, or age.

### Pupillometric Data – Exploratory Model

Participants with higher ADHD symptoms, regardless of diagnostic group, showed higher PD peak amplitude (*B* = 0.003, *p* = .01, [0.001, 0.005]) and longer latency to PD peak (*B* = 15.49, *p* < .001, [9.02, 21.96]). While there was no effect of higher trait anxiety on PD peak amplitude (*p* > .1), participants with higher trait anxiety showed shorter latency to PD peak (*B* = -11.85, *p* = .001, [-18.87, -4.83]). No effects of state anxiety or sex on PD peak amplitude and latency to PD peak were found.

### Pupillometric Data – Cluster-Based Permutation Test

**Fig. 5:**
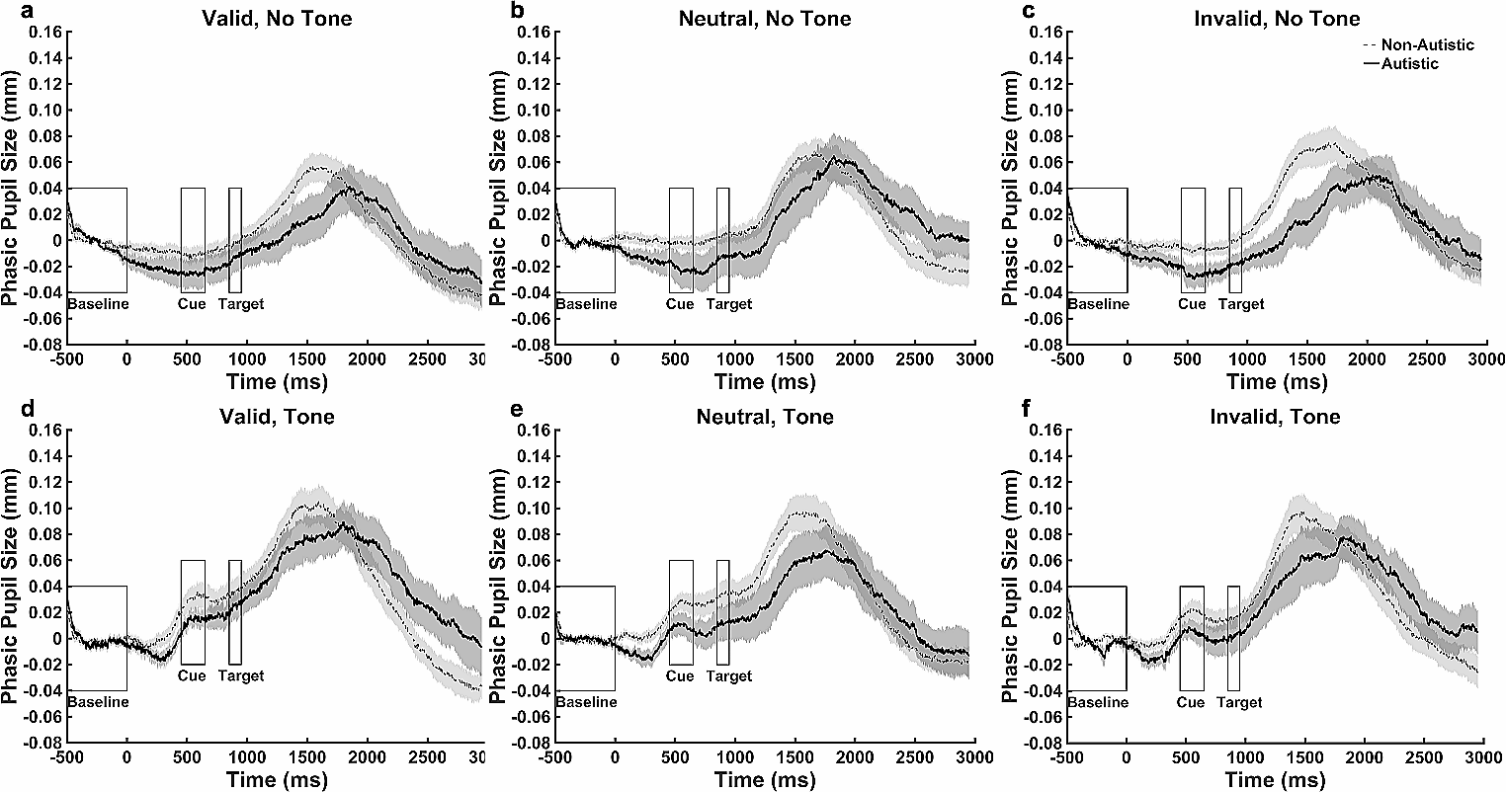
Mean Phasic Pupil Size During the Exogenous Task by Cue Type. *Note* The change of pupil size over the exogenous orienting trial was shown in each cue and tone condition. Baseline pupil size = the average pupil size over the 500 ms period before the onset of any stimuli. Phasic pupil size = pupil sizes during the trial - the baseline pupil size. In the tone condition, an alerting tone (300 ms) was played at time zero. In the no-tone condition, no stimulus was shown until the onset of a cue. The cue (200 ms) was presented at the 450 ms and the target (100 ms) was presented at the 850 ms. The dashed line represents the non-autistic group. The solid line represents autistic group. Data are expressed as mean ± standard errors

Figure 5 shows the progression of mean phasic pupil size over the course of different types of trials. A cluster-based permutation test was conducted between groups for the no-tone valid and invalid trials and there were no significant group differences (*p* ≥ .05 for all clusters).

### Endogenous Task

#### Behavioural Data–Overall Performance

Response time, accuracy, and the number of incorrect responses, omission errors, and anticipation errors in the autistic and non-autistic group were reported in Table 3.

**Table 3:** Response Time, Accuracy, and the Number of Errors in the Autistic and Non-Autistic Groups in the Endogenous Task

Variable	Autistic M (SD)	Non-autistic M (SD)
Response Time (ms)		
Valid	349 (92)	352 (118)
Neutral	376 (83)	397 (156)
Invalid	401 (122)	392 (131)
Accuracy (%)		
Valid	97 (4)	97 (4)
Neutral	97 (5)	98 (5)
Invalid	90 (12)	86 (19)
Incorrect responses	5 (5)	6 (7)
Omission errors	1 (3)	1 (2)
Anticipation errors	1 (4)	1 (3)

#### Behavioural Data–Base Model

##### Group

**Fig. 6:**
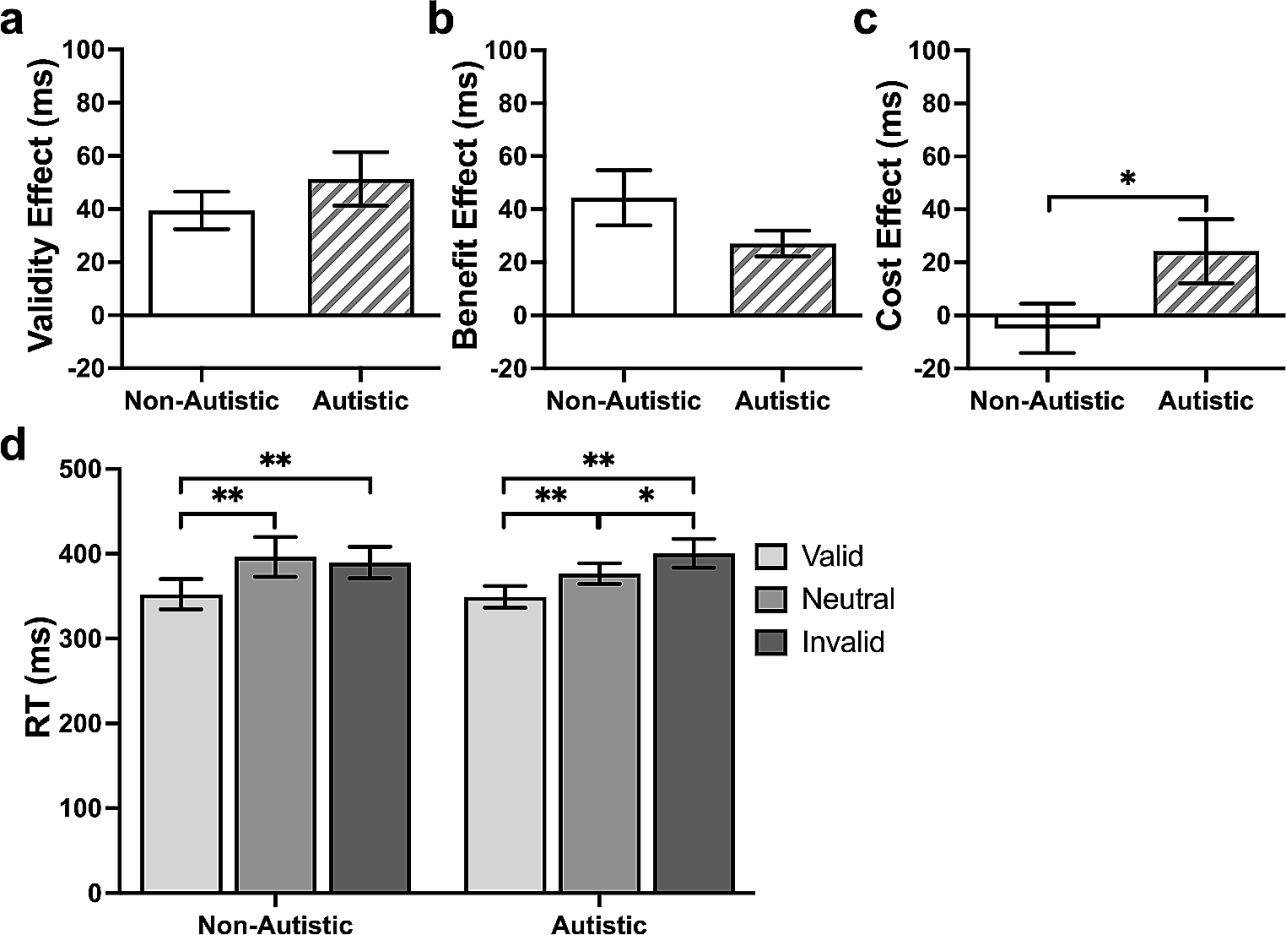
Cueing Effects and Mean Response Time (RT) in the Endogenous Task. *Note* (**a**) Non-significant group difference in validity effect (invalid − valid trials RT). (**b**) Non-significant group difference in benefit effect (neutral − valid trials RT). (**c**) Significant group difference in cost effect (invalid − neutral trials RT). (**d**) Differences of RT between each cue conditions in each group. Data are expressed as mean ± standard errors. *p < .05, **p < .001

The autistic group showed a larger cost effect (*B* = 32.66, *p* = .04, [0.95, 64.37]; Fig. 6c) but comparable validity (*B* = 16.07, *p* = .19, [-7.70, 39.86]; Fig. 6a) and benefit effects (*B* = -16.50, *p* = .12, [-37.27, 4.25]; Fig. 6b) relative to the non-autistic group. No group differences were found in the valid (*p* = .89), invalid (*p* = .53), or neutral (*p* = .69) trials. Both groups responded more slowly in the invalid compared to valid trials (both *p*s < 0.001) and more slowly in the neutral compared to valid trials (both *p*s < 0.001). Only autistic participants, however, responded more slowly in the invalid compared to neutral trials (*p* = .002; Fig. 6d)

##### Alerting Tone

**Fig. 7:**
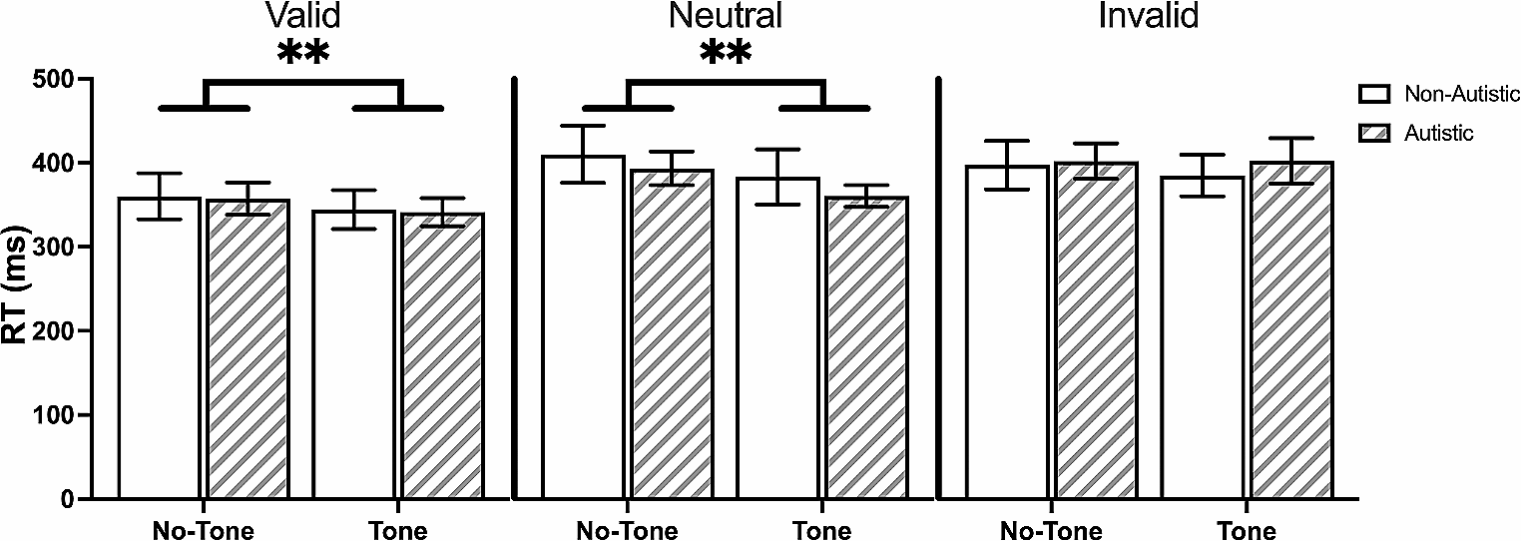
Effects of Alerting Tone on Mean Response Time (RT) in the Endogenous Task. *Note* The alerting tone quickened response time in the exogenous task in the valid and neutral trials, but not in the invalid trials. There were no significant group differences. Solid-coloured bars represent non-autistic group. Striped bars represent autistic group. Data are expressed as mean ± standard errors. **p < .001

The alerting tone was associated with a decreased benefit effect (*B* = -14.08, *p* = .02, [-26.18, -1.98]) and increased cost effect (*B* = 24.85, *p* = .01, [5.99, 43.79]), but did not affect the validity effect (*B* = 10.63, *p* = .15, [-3.83, 25.09]), for both groups. When the alerting tone was present, participants responded more quickly in the valid and neutral trials (both *p*s < 0.001) but similarly in the invalid trials (*p* = .44; Fig. 7)

##### Age

**Fig. 8:**
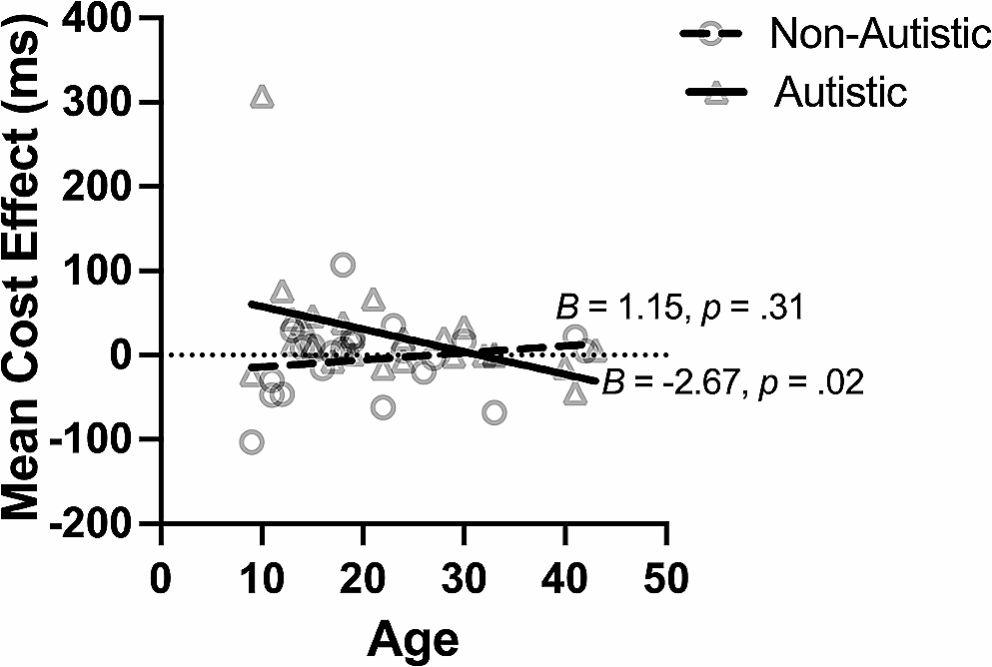
Relationship Between Cost Effect and Age in the Endogenous Task. *Note* The cost effect decreased with older age in the autistic group (solid line), but not in the non-autistic group (dashed line). The circle represents individual non-autistic participants. The triangle represents individual autistic participants

There was a significant interaction between age and group for the benefit effect (*B* = 2.17, *p* = .048, [0.02, 4.32]), but follow-up analyses did not suggest a group difference. Post hoc analyses indicated that the benefit effect was not associated with age in either group (autistic: *B* = 0.76, *p* = .11, [-0.16,1.68]; non-autistic: *B* = -1.41, *p* = .19, [-3.55, 0.72]). There was also a significant interaction between age and group for the cost effect (Age × Group: *B* = -3.82, *p* = .02, [-7.07, -0.58]). For the autistic participants, cost effect decreased with increasing age (*B* = -2.67, *p* = .02, [-4.93, -0.41]; Fig. 8), with faster responses in the invalid (*p* = .03) but not neutral (*p* = .17) trials. For the non-autistic participants, the cost effect was not affected by age (*B* = 1.15, *p* = .31, [-1.07, 3.37]). No effect of age on the validity effect was found for either group (*B* = -1.22, *p* = .06, [-2.48, 0.05])

#### Behavioural Data–Exploratory Model

Sex, ADHD symptoms, state anxiety, and trait anxiety did not affect any of the outcome variables and did not affect the groups differently.

### Pupillometric Data–Base Model

PD peak amplitude was lower in the valid compared to neutral (*B* = -0.03, *p* = .001, [-0.04, -0.01]) and invalid conditions (*B* = -0.03, *p* < .001, [-0.05, -0.02]); no difference was noted between the neutral and invalid conditions (*B* = 0.01, *p* = .51, [-0.01, 0.02]). PD peak amplitude was larger in the tone compared to no-tone condition (*B* = 0.04, *p* < .001, [0.02, 0.05]). PD peak amplitude did not differ between the groups and was not affected by age.

The latency to PD peak was not affected by group, cue condition, tone condition, or age.

#### Pupillometric Data–Exploratory Model

PD peak amplitude was not affected by sex, ADHD symptoms, state anxiety, or trait anxiety.

Latency to PD peak was shorter in females compared to males (*B* = -199.16, *p* = .02, [-362.84, -35.48]) and was longer in participants with higher ADHD symptoms (*B* = 8.78, *p* = .04, [0.40, 17.16]). No effects of state or trait anxiety on latency to PD peak were found.

### Pupillometric Data–Cluster-Based Permutation Test

**Fig. 9:**
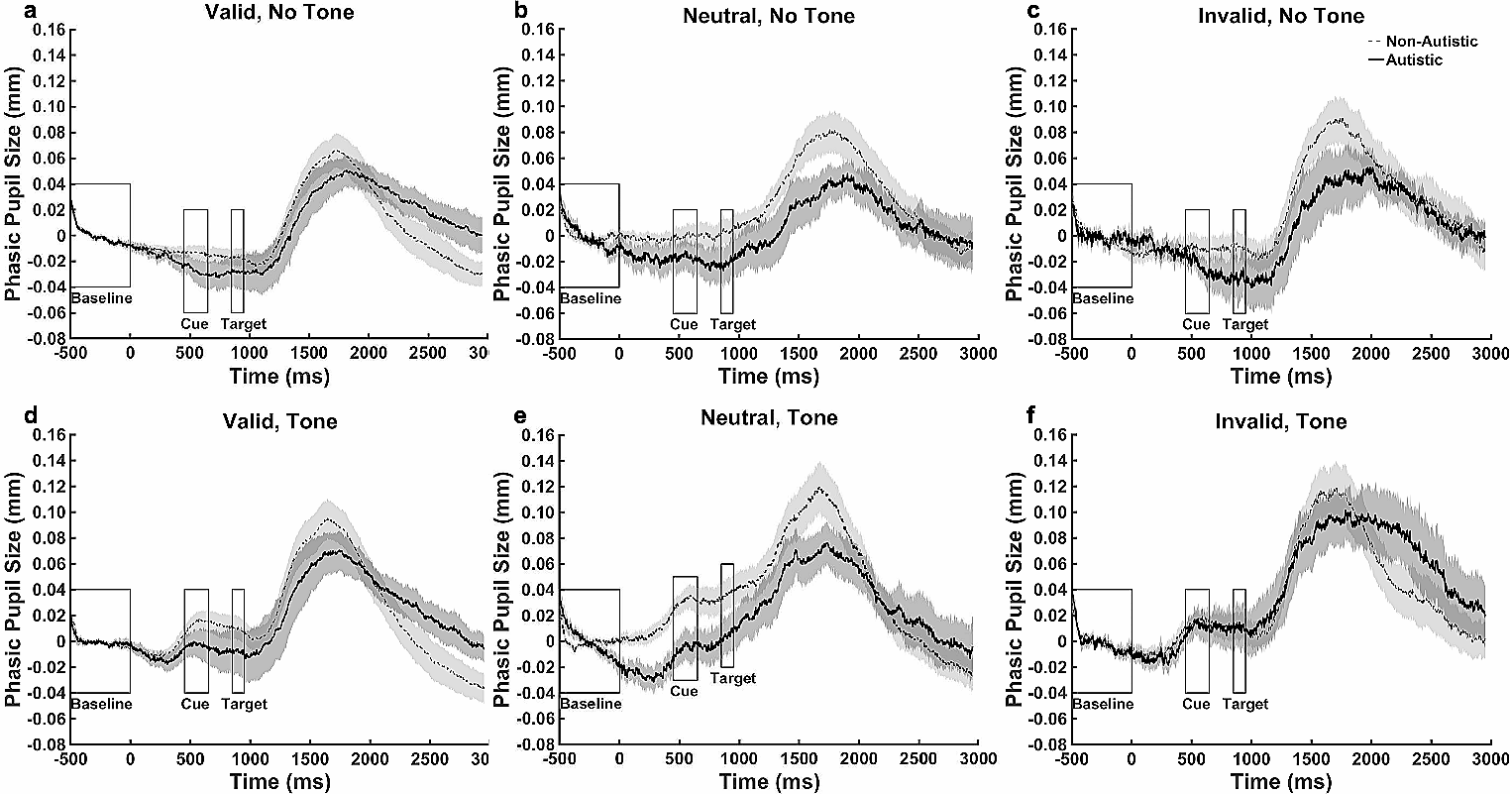
Mean Phasic Pupil Size During the Endogenous Task by Cue Type. *Note* The change of pupil size over the endogenous orienting trial was shown in each cue and tone condition. Baseline pupil size = the average pupil size over the 500 ms period before the onset of any stimuli. Phasic pupil size = pupil sizes during the trial - the baseline pupil size. In the tone condition, an alerting tone (300 ms) was played at time zero. In the no-tone condition, no stimulus was shown until the onset of a cue. The cue (200 ms) was presented at the 450 ms and the target (100 ms) was presented at the 850 ms. The dashed line represents the non-autistic group. The solid line represents autistic group. Data are expressed as mean ± standard errors

Figure 9 shows the progression of mean phasic pupil size over the course of different types of trials. The cluster-based permutation test was conducted between groups during the no-tone valid and invalid trials and showed no group differences (*p* ≥ .05 for all clusters).

## Discussion

Two types of attention orienting in autistic people were examined with consideration of several potential confounders. Autistic participants demonstrated larger validity and benefit effects in the exogenous orienting task and a larger cost effect in the endogenous orienting task compared to non-autistic participants, suggesting efficient exogenous orienting and inefficient endogenous orienting. Atypical attention orienting in autism was associated with age and co-occurring symptoms of ADHD and anxiety. No group differences were noted for alertness or sex. Through careful use of cues to evoke the exogenous and endogenous orienting systems separately, here we have clarified specific attention orienting differences associated with autism and highlighted the importance of age and the effects of two common co-occurring symptoms.

Autistic people demonstrated superior exogenous orienting, with faster responses to valid versus neutral cues compared with the non-autistic group, which is a novel finding. One difference with previous studies (e.g., Ronconi et al., 2018) was our use of a non-predictive peripheral cue without additional interpretation or discrimination of the stimuli, providing a more accurate measurement of exogenous orienting. Consistent with the current finding, the ventral attention network, which modulates stimulus-driven orienting, was found to show stronger connectivity in autistic people (Fitzgerald et al., 2015).

Autistic people showed inefficient endogenous orienting, with slower responses to invalid versus neutral cues compared with the non-autistic group. This result suggests that autistic people are less efficient at voluntarily disengaging attention from the invalidly cued to the actual target location. Although irregular attention disengagement in ASD has previously been reported (e.g., R. Landry & Bryson, 2004), this study is the first to use non-social rule-based cues, providing strong support that inefficient disengagement in autistic people is related to irregular voluntary attention. In line with our finding, the dorsal attention network, which modulates goal-driven orienting, has been reported to show weaker functional connectivity in autistic people (Fitzgerald et al., 2015).

The contrasting performance between exogenous and endogenous tasks in autistic people challenges previous proposals that sticky attention (R. Landry & Bryson, 2004; Rincover & Ducharme, 1987) or impaired attention disengagement (Keehn et al., 2013) is a general characteristic of ASD. Rather, autistic people may only demonstrate deficits in attention disengagement when high-level cognitive control is required, such as in our endogenous orienting task.

The alerting tone did not exert a differential effect on response times or pupillometric responses between the groups. These findings suggest that autistic people were able to spontaneously regulate their alertness during attention orienting. Boxhoorn et al. (2020) found an association between larger pupil dilation and slower orienting in autistic participants, suggesting a negative effect of hyperalert status. Boxhoorn et al. (2020), however, required participants to distinguish the target from a distractor. Increased pupil dilation in the autistic group may have reflected greater mental effort to discriminate stimuli and control responses. Likewise, interpreting social cues was associated with atypically larger pupil dilation in autism (Aldaqre et al., 2016). Atypical attention orienting per se may not be explained by atypical alertness in autistic people.

ADHD symptoms contributed to difficulty in disengaging exogenous attention, in both groups. This may be due to reduced ability to suppress incorrect or distracting information in people with high ADHD symptoms (Brocki et al., 2007; Mullane et al., 2009; Polner et al., 2015). No effect of ADHD symptoms was observed in the endogenous task, which may be because endogenous orienting involves higher cognitive control, requires participants to focus, and potentially reduces inattentive tendencies. As autistic people are more likely to demonstrate elevated ADHD symptoms than non-autistic people in the general population (Das et al., 2012; Lecavalier et al., 2019), co-occurring ADHD symptoms should be considered an important factor that may heighten atypical attention orienting in autism.

Higher symptoms of trait anxiety were associated with faster disengagement of exogenous attention, but only in the autistic group. One explanation is that both ASD and anxiety are associated with heightened sensitivity to the sudden onset of salient stimuli (Berggren et al., 2015; Kopec et al., 2020; Moser et al., 2012), speeding the detection of the target after an invalid cue. As the non-autistic group showed lower trait anxiety symptoms, the association between anxiety and exogenous orienting was lessened in this group. It is noted that no effect of trait anxiety was observed on endogenous orienting in either group. Endogenous orienting is a goal-driven process and is not dependent on stimulus saliency, so it may not be as susceptible as exogenous orienting to the influences of anxiety (Moriya & Tanno, 2009).

As age increased, participants demonstrated faster responses in all trial types with a smaller orienting and cost effect in the exogenous task, suggesting an age-dependent improvement in both autistic and non-autistic groups. An age effect on exogenous orienting was not found in another ASD study (Zhao et al., 2016), possibly because the previous study included a narrower age range (only adolescents) compared to the current study. In the endogenous orienting task, older autistic people showed a smaller cost effect, an effect not seen in the non-autistic group. This may reflect specific compensatory mechanisms for endogenous orienting in autistic people. Previous neuroimaging studies have observed that autistic people exhibit greater inter-regional brain connectivity relative to non-autistic people during endogenous orienting, reflecting alternative or effortful attention processing to compensate for the deficient attention orienting (Belmonte & Yurgelun-Todd, 2003; Fitzgerald et al., 2015).

Regarding limitations, first, the autistic participants were adolescents and adults with average non-verbal visual intelligence. The current results are not representative of all individuals on the spectrum. Nevertheless, as most of the previous autism research has focused on children and males, the current study brings valuable insight into attention functioning in older and female autistic people. Second, the conclusions drawn are limited by the large age range (9–43 years) within the sample. Studies with larger sample sizes or with the adoption of a longitudinal design are needed to further evaluate the developmental trajectory of attention orienting in autistic people. Third, arousal levels may not be homogenous in autism, with some showing hypo-arousal and others hyper-arousal (Schoen et al., 2008). The lack of differential alerting effects in the autistic group may be because different profiles of arousal were averaged in the group analysis. The sample size was not large enough to categorise the autistic group into hyper- and hypo-arousal subgroups for further examination.

In conclusion, the current study showed that autistic people were easily attracted by external stimuli and were slower at voluntarily disengaging attention, providing new evidence of superior exogenous and inefficient endogenous orienting in autism. In the examination of potential confounders, atypical attention orienting in autistic people was more likely to be observed at a younger age and could be moderated by co-occurring ADHD and anxiety symptoms but was not affected by alertness or sex. In summary, the current study offered new insight into the attention profile of autism and demonstrated the importance of considering the effect of age and co-occurring symptoms in the assessment of attention orienting in autism. Given the fundamental role of attention in cognition, the atypical characteristics of attention orienting identified in the current study are likely to contribute to a variety of differences in social and cognitive functions in autism, which is worth exploring in future studies.

## Data Availability

The research data are available at Open Science Framework (DOI: 10.17605/OSF.IO/NGQT2).
